# The subresolution DaTSCAN phantom: a cost-effective, flexible alternative to traditional phantom technology

**DOI:** 10.1097/MNM.0000000000000801

**Published:** 2018-01-17

**Authors:** Jonathan C. Taylor, Nicholas Vennart, Ian Negus, Robin Holmes, Oliver Bandmann, Christine Lo, John Fenner

**Affiliations:** Departments of a Nuclear Medicine; b Neurology, Sheffield Teaching Hospitals NHS Foundation Trust; c Department of Infection, Immunity and Cardiovascular Disease (IICD), University of Sheffield, Insigneo, Royal Hallamshire Hospital; d Department of Neuroscience, Sheffield Institute for Translational Neuroscience, University of Sheffield, Sheffield; e Department of Medical Physics, Gateshead Health NHS Foundation Trust, Queen Elizabeth Hospital, Gateshead; f Department of Medical Physics and Bioengineering, University Hospitals Bristol NHS Foundation Trust, Bristol Royal Infirmary, Bristol, UK

**Keywords:** ^123^I-FP-CIT, Parkinson’s disease, phantom, semi-quantification

## Abstract

The Alderson striatal phantom is frequently used to assess ^123^I-FP-CIT (Ioflupane) image quality and to test semi-quantification software. However, its design is associated with a number of limitations, in particular: unrealistic image appearances and inflexibility. A new physical phantom approach is proposed on the basis of subresolution phantom technology. The design incorporates thin slabs of attenuating material generated through additive manufacturing, and paper sheets with radioactive ink patterns printed on their surface, created with a conventional inkjet printer. The paper sheets and attenuating slabs are interleaved before scanning. Use of thin layers ensures that they cannot be individually resolved on reconstructed images. An investigation was carried out to demonstrate the performance of such a phantom in producing simplified ^123^I-FP-CIT uptake patterns. Single photon emission computed tomography imaging was carried out on an assembled phantom designed to mimic a healthy patient. Striatal binding ratio results and linear striatal dimensions were calculated from the reconstructed data and compared with that of 22 clinical patients without evidence of Parkinsonian syndrome, determined from clinical follow-up. Striatal binding ratio results for the fully assembled phantom were: 3.1, 3.3, 2.9 and 2.6 for the right caudate, left caudate, right putamen and right caudate, respectively. All were within two SDs of results derived from a cohort of clinical patients. Medial–lateral and anterior–posterior dimensions of the simulated striata were also within the range of values seen in clinical data. This work provides the foundation for the generation of a range of more clinically realistic, physical phantoms.

## Abstract 2

Supplemental Digital Content is available in the text.

## Introduction

^123^I-FP-CIT (DaTSCAN) is a gamma camera single photon emission computed tomography (SPECT) imaging procedure for assessment of the function of the nigrostriatal pathway, for example, to distinguish between essential tremor and idiopathic Parkinson’s disease.

Typically, diagnostic assessment involves both visual analysis and semi-quantitative measurements of relative tracer uptake in striatal regions compared with a reference brain region. Images acquired from phantoms, where the true concentration of radioactive tracer is known, are often used to validate and test semi-quantification software tools. This can be particularly important for comparing different cameras, collimators, acquisition protocols and reconstruction methods, all of which can have a significant impact on quantification results 1–6 and therefore potentially on patient care. ^123^I-FP-CIT phantoms are also used to compare image quality between different hardware and to optimize reconstruction protocols.

**Fig. 1:**
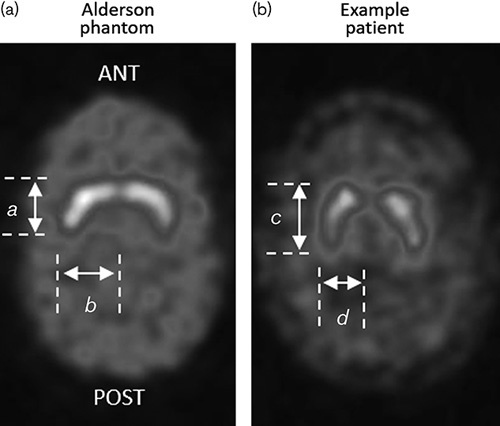
Reconstructed, central transaxial slice from a typical normal patient (b) and from the Alderson phantom (a), demonstrating clear differences in striatal shape (*a*/*b*<*c*/*d*). In this case the Alderson phantom was filled with an 8 : 1 striatum to reference brain activity concentration ratio. Each slice is scaled to its maximum pixel value.

A phantom that is frequently used for these purposes is the Alderson striatal phantom (Radiology Support Devices, *http://www.rsdphantoms.com/*). It consists of separate fillable cavities for the left and right putamen and caudate, and a large region encompassing the remaining brain. The major advantage of this design is that it is reproducible, because of the rigid, fixed cavity walls. However, it suffers from a number of limitations. First, for an anthropomorphic phantom, it lacks fidelity as the shape of the putamen and caudate sections do not resemble that of most clinical patients. Consequently, in reconstructions, the phantom gives a striatal shape that appears to extend more in the medial–lateral direction than in the anterior–posterior direction. This is different to patterns typically seen for normal patients, whose striata usually extend more in the anterior–posterior direction. Figure 1 shows these contrasting image appearances using example data acquired at our institution. Acquisition equipment, reconstruction settings and head alignment were consistent in both the examples shown. For semi-quantification software that relies on automated image registration, these discrepancies in appearance could bias the positioning of regions of interest, which may have a significant effect on results.

The inflexibility of the Alderson design is also a disadvantage. The geometry of the phantom is fixed to an idealized anatomical shape. Recent research based on simulated acquisitions has suggested that differences in striatal anatomy can have an impact on semi-quantification results 7. A new type of physical phantom is required to support investigation of such findings.

This study describes the application of an established technology, subresolution sandwich phantoms (SSPs) 8–10, for creating flexible, ^123^I-FP-CIT phantom images, which can potentially overcome these limitations. SSPs are created through the assembly of layers of paper sheets, which have radioactive ink patterns printed on their surface. These are interleaved between slabs of attenuating material. The ink patterns are created by a standard inkjet printer, using a (black) cartridge containing both printer ink and aqueous radioactive solution. The concentration of radioactive ink solution that is printed per unit area depends on the printer’s installed ink profile curve, which provides a lookup table for converting input pixel intensities into printed ink concentrations. In the context of the gamma camera this enables a bespoke activity distribution to be generated for assessment of imaging performance.

SSPs have already been used successfully for a range of applications, including simulation of SPECT brain perfusion scans 8–10 and PET scans 11. This technical note presents SSP methodology in the context of ^123^I-FP-CIT imaging and describes a novel method for generating an anatomical print template. A fully assembled phantom is compared with patient data through semi-quantitative analysis and through measurements of the shape of the striata.

## Materials and methods

The following method describes phantom production (radionuclide printing and assembly), followed by a study of its efficacy through gamma camera imaging. The printer used was a commercially available inkjet device (HP 8100 officejet pro, Hewlett-Packard, Palo Alto, California, USA). Pixel greyscale values are reported on a linear scale from 0.0 (white) to 1.0 (black). All values between 0.0 and 1.0 represent different shades of grey as dictated by the printer profile curve.

### Printer adaptation and performance

Before producing SSPs the ink profile curve must be established in order to deliver the desired mapping between pixel intensity of the input template and printed ink density. This was determined by printing technetium-99m pertechnetate and ink solution at 12 greyscale levels in small rectangular shapes (2 cm×5 cm), on standard office paper (80 g/m^2^). Each was cut out and measured within a Perkin-Elmer 2480 sample counter (Perkin-Elmer, Waltham, Massachusetts, USA) to determine the output count level. The procedure was repeated five times. Results were used to inform the anatomical template design and are summarized in supplementary data (Supplementary Fig. A, Supplemental digital content 1, *http://links.lww.com/NMC/A124*).

### Phantom structure and assembly

**Fig. 2:**
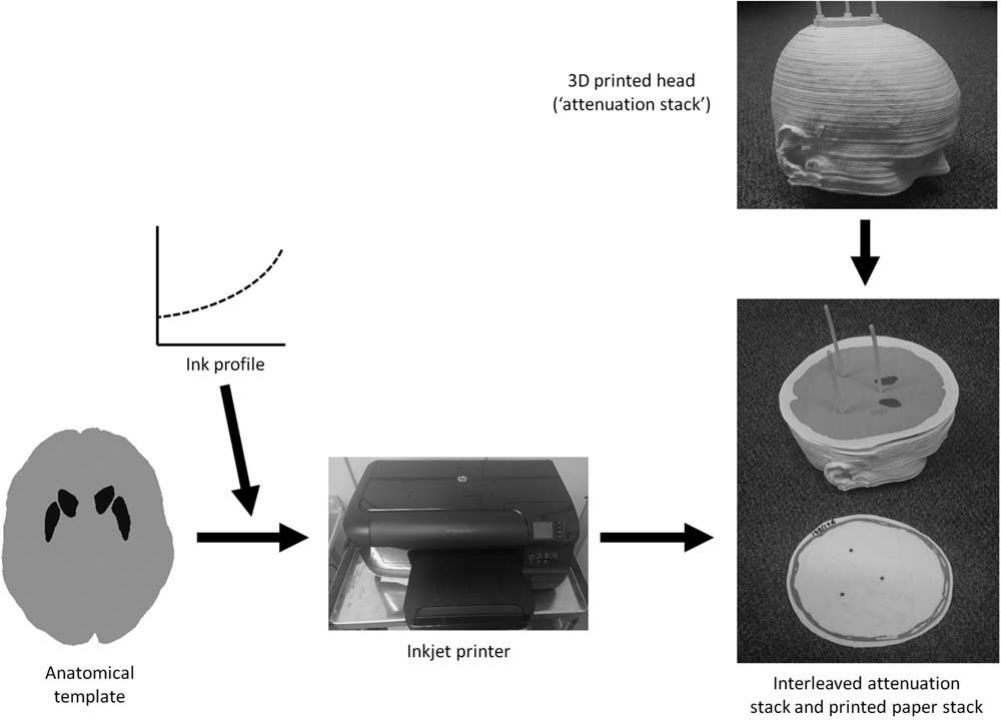
Workflow depicting the subresolution sandwich phantom manufacturing process.

The SSP technique requires that a stack of printed radioactive sheets are placed within a solid structure that maintains the correct positioning of the sheets with respect to each other but also provides an attenuating medium, similar to that of the human body. Previous studies have used stacked plastic layers for this purpose 10,11 or three-dimensional (3D) printed layers 12. In this work a 3D printed head structure was used, created at University Hospitals Bristol (Bristol, UK) to mimic the shape and scatter properties of a real human head (Fig. 2). The design and manufacture of this structure was similar to that described in a previous publication by the same group 12, utilizing the same Fused Deposition Modelling technology.

Briefly, the head comprised a series of 1.9 mm thick 3D printed slabs, whose geometry was taken from a computed tomography (CT) scan of a patient, after segmentation into bone and soft tissue compartments using SPM software (SPM 12, *http://www.fil.ion.ucl.ac.uk/spm/software/spm12/*), and reslicing in the transaxial plane. The soft tissue compartment was manufactured from standard polylactic acid filament printed at 85% infill density and the bone compartment (i.e. the skull) was created using polylactic acid doped with bronze (20% by weight). The printed soft tissue structure had a linear attenuation coefficient of 0.168/cm and the bone structure a linear attenuation coefficient of 0.225/cm, both at 140 keV. Three holes were embedded throughout the head design to accommodate 4 mm diameter, nylon guide rods. These acted as guides for placement of the paper sheets, interleaved with single 3D printed slabs, and held the structure together. The spacing between printed paper sheets was less than half the extrinsic resolution of a standard gamma camera such that the different layers could not be distinguished in the final image.

### Phantom template shape and printing

The phantom required that the geometry and radionuclide distribution of the simulated patient be defined. For the purposes of this work a simple anatomical design was chosen on the basis of a well-established MRI dataset, the Montreal Neurological Institute (MNI) template 13. Grey and white matter and cerebrospinal fluid regions were generated in SPM software by segmenting the T1-weighted MNI data. These three regions were combined to give a simplified, uniform area of uptake encompassing the whole brain. The Automated Anatomical Labelling atlas 14 is a widely used parcellation of the MNI template. The regions depicting the left and right putamen and caudate were chosen for this study as a means of defining the boundaries of the striatal uptake volume. The striatal and whole brain regions were combined within Matlab software (Matlab, Natick, Massachusetts, USA) and then resampled to a transaxial slice thickness of 2 mm to generate the final anatomical ^123^I-FP-CIT template.

**Fig. 3:**
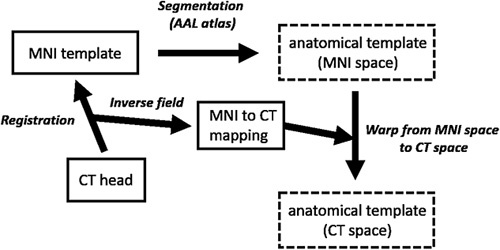
Workflow depicting the steps taken to create an anatomical ^123^I-FP-CIT template, fitted to the geometry of the attenuation stack (based on a patient’s CT scan). AAL atlas, Automated Anatomical Labelling atlas; CT, computed tomography; MNI, Montreal Neurological Institute.

In order that the derived anatomical slices would fit the geometry of the 3D printed head structure, image registration was required. SPM was used to segment a rewindowed version of the CT scan, originally used to create the attenuation stack. This procedure segments and spatially normalizes images to MNI space and enables the creation of forward and inverse deformation fields 15. The inverse deformation field was then used to map the anatomical template from MNI space on to the CT scan (see flowchart in Fig. 3).

### Gamma camera study

SPECT imaging was performed to compare a fully assembled phantom with patient data. All tests were performed on a GE Infinia gamma camera (GE Healthcare, Chicago, Illinois, USA), equipped with dual low energy high resolution collimators. Greyscale levels for the print template were chosen on the basis of the results of the ink profile curve measurements obtained for technetium-99m ink solution. Because of paper saturation effects for greyscale levels of 0.95 and 1.0 (leading to increased variability; Supplementary Fig. A, Supplemental digital content 1, *http://links.lww.com/NMC/A124*), the maximum greyscale value used was 0.9. This was assigned to all pixels within the striatum. For the remainder of the brain a lower greyscale value was used, derived from the ink profile curve (Supplementary Fig. A, Supplemental digital content 1, *http://links.lww.com/NMC/A124*).

Each slice of the anatomical template was printed from an ink cartridge containing both ^123^I iodide (at a concentration of 37 MBq/ml) and black ink, in a 1 : 1 volume ratio, giving an overall radioactive concentration in the cartridge of ~18 MBq/ml. The total volume in the cartridge was ~16 ml. Pixel values were set to give a striatum to brain count density ratio of 8 : 1, reflective of a normal patient 16,17.

SPECT acquisition parameters for the fully assembled phantom were similar to those used clinically in the local department. Sixty projections were collected over 180°, per detector. A zoom of 1.2 was applied, with an energy window of 159 keV±10%. The radius of rotation was set at 14 cm. Scan time was adjusted to achieve a total count over the length of the acquisition of greater than 1.5 Mcts.

Reconstruction was performed using an Xeleris workstation (version 2.1, GE Healthcare, Chicago, Illinois, USA), using standard clinical parameters [ordered subset expectation maximization with two iterations and 10 subsets and a Butterworth postfilter (power 10, cut-off 0.7 cycles/cm)]. Reconstructed pixel size was 3.68 mm. The reconstructed data were passed to BRASS software, version 2.5 (Hermes Medical Solutions, Stockholm, Sweden) to quantify striatal binding ratios within the putamen and caudate on both sides as compared with a reference region in the remainder of the brain. In addition, the medial–lateral extent and the anterior–posterior extent of the striata in the new SSP design were measured on a two-dimensional slab that was created through summation of 10 central brain slices. Ratios of these two values provided a simple measure of striatal shape. This analysis was carried out in MIM software (MIM Software, Beachwood, Ohio, USA) after manually aligning images such that the transaxial plane was parallel to the line connecting the anterior and posterior commissure, and then further adjusting alignment such that there was approximate symmetry between left and right hemispheres in both coronal and transaxial views. Linear measurements of left and right striata were carried out manually with the caliper tool.

Analysis methods were also applied to reconstructed images from 22 clinical patients, each of which was confirmed (with high probability) as not having a Parkinsonian syndrome through clinical follow-up subsequent to ^123^I-FP-CIT imaging (mean time of follow-up was 33 months). Patients were imaged under similar acquisition conditions as the phantom, using either the same gamma camera or a closely related model from the same manufacturer. Ethical approval was granted by City and East Research Ethics Committee for use of the historical patient data in this way.

## Results

Approximately 4 ml of the combined ^123^I iodide and black ink solution was required to print 56 slices of the ^123^I-FP-CIT template, covering the entire brain. Printing was completed within 30 min and phantom assembly took ~45 min. To acquire 1.5 Mcts over the course of the acquisition an imaging time of 30 s/projection was used.

**Fig. 4:**
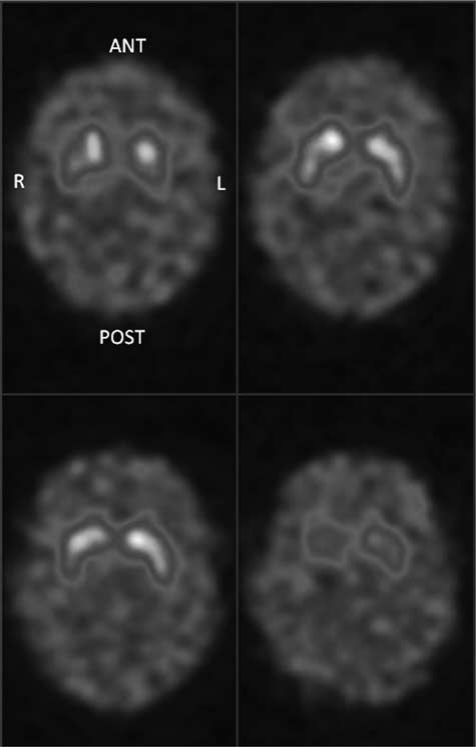
Four consecutive, reconstructed, transaxial slices from the phantom. Images are scaled to the maximum pixel value within the four slices. Slice thickness=7.2 mm. L, left; R, right.

Figure 4 shows transaxial, reconstructed slices from the SSP acquisition and Table 1 shows striatal binding ratio results for these images. Results were generated in BRASS using automatic registration and processing settings. For comparison, Table 2 shows semi-quantification results from 22 clinical patients without evidence of dopaminergic deficit. The same processing settings were used as for the phantom.

**Table 1:**
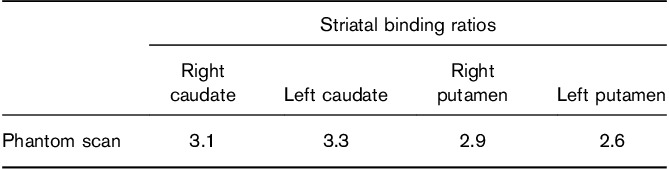
Striatal binding ratio results from the fully assembled phantom

**Table 2:**
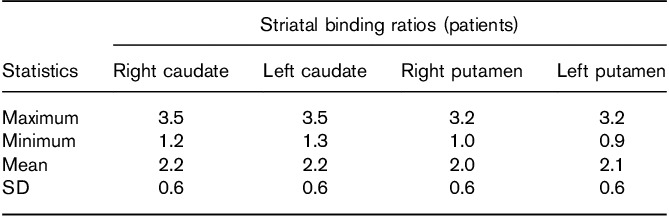
Summary striatal binding ratio statistics for 22 clinical patients without a Parkinsonian syndrome

**Fig. 5:**
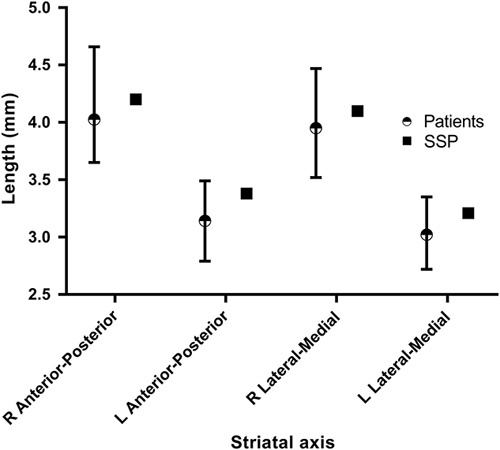
Linear measurements of the striatum in images acquired from the phantom and from a group of 22 patients without evidence of dopaminergic deficit. Whiskers represent maximum and minimum lengths. L, left; R, right; SSP, subresolution sandwich phantoms.

**Fig. 6:**
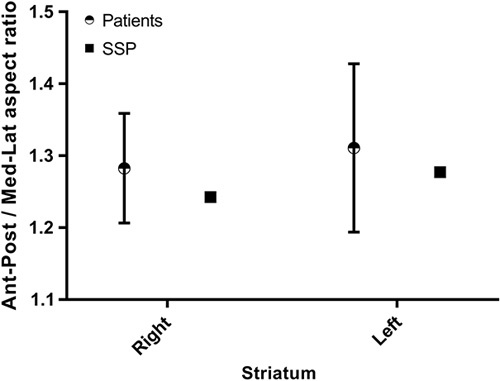
Anterior–posterior/medial–lateral aspect ratio measurements from the phantom and a group of 22 patients without evidence of dopaminergic deficit. Whiskers represent maximum and minimum aspect ratios. SSP, subresolution sandwich phantoms

Figure 5 and 6 show linear measurements of striatal geometry, as well as derived anterior–posterior/medial–lateral aspect ratio measurements from the phantom and the group of 22 patients.

## Discussion

Although increasingly used, the SSP technique has not previously been adapted for the radionuclide ^123^I and has not yet been applied to simulation of ^123^I-FP-CIT images. This work shows a straightforward method for generating an anatomical template, which was printed, interleaved between attenuation slabs, scanned and compared with clinical patient data.

The volume of ink solution used suggests that ~5 full phantoms could be printed from a single standard-sized cartridge (if filled to a maximum capacity of 23 ml), which should be sufficient for many applications. The total printing and assembly time was longer than the preparation time required for the Alderson phantom (typically up to 1 h). However, the SSP assembly process has not yet been optimized. There is potential for time savings by, for example, rounding off the ends of the nylon guides rods to enable quicker stacking of paper and plastic layers.

Utilizing an ink printer presents an increased risk of radioactive contamination from ink droplets, particularly during phantom assembly where individual paper sheets must be manually placed over guide rods. Printing and assembly were therefore conducted in a lab environment with appropriate controls in place (including preventing access to other staff). However, the total activity present in ink droplets (and in each printed sheet) is likely to be very low. Furthermore, no contamination was measured on the individuals constructing the phantom after assembly was completed, or on the surfaces next to the printer.

The imaging time required to reach typical clinical count levels was similar to that of patients, which suggests that even when using ^123^I iodide at a relatively low radioactivity concentration (37 MBq/ml), SSP is a practical alternative technology to traditional fixed cavity phantoms. This is an important finding as ^123^I iodide is widely (and cheaply) available to UK Nuclear Medicine departments.

Visual analysis of the reconstructed phantom images (Fig. 4) shows uniform, high uptake throughout the striata on both sides with a wide area of relatively low, uniform uptake in remainder of the brain. This closely reflects the characteristics of the simplified anatomical template and provides some reassurance that there were no significant problems in phantom assembly. This also reflects findings from previous tests of printer performance, where single slices from the anatomical template were printed out and imaged at the camera face with a static two-dimensional acquisition. Here, threshold-based segmentation of the template slice and acquired image gave regions of interest with a high degree of overlap in both the striatal structures and the whole brain. For example, the mean Dice coefficient for the whole brain was 0.99 across five slices in the centre of the head (where Dice coefficient is a measure of the ratio of the area of intersection between two regions of interest in the different images, as compared with the total combined area). These results suggest that phantoms produced by the described technique are a good representation of the selected anatomical template.

The reconstructed phantom images gave striatal binding ratio results (in the left and right putamen and caudate) that were above the mean but still well within the range of results generated for a group of 22 non-Parkinsonian patients, validating the fidelity of the SSP method in producing image features reflective of a particular patient cohort. In addition, linear measurements of the shape and size of the striata were also within the range measured from real patients. This is reassuring in light of criticisms of the Alderson phantom, which produces reconstructed images that appear overly elongated in medial–lateral direction as compared with the anterior–posterior direction. Indeed, subsequent analysis of a previously acquired Alderson phantom dataset, also having a 8 : 1 radioactivity concentration ratio, produced left and right aspect ratio results of 1.0 and 1.0, respectively, outside the range reported for the 22 patients (Fig. 6).

However, these results should be interpreted in the light of methodological limitations. First, patient age is a known covariate for striatal binding ratio 16 and was ignored in the comparison exercise. Second, the image analysis methods selected do not allow for a comprehensive assessment of the ability of the SSP design to replicate normal patient appearances. Indeed, visual inspection of the reconstructed SSP data (Fig. 4) suggests that appearances are still overly simplified as compared with real patient data. Although these limitations dictate that results benefit from conservative interpretation, nonetheless the findings indicate suitability of the method for investigations of derived ^123^I-FP-CIT image features, such as binding ratios or striatal shape measures.

This study used a bespoke 3D printed head geometry to provide the attenuating medium. Reproducing this head is not straightforward. However, similar results are likely to be possible with more simple attenuating materials and shapes, such as layers of poly(methyl methacrylate) in a uniform ellipsoid shape, as has been used previously in the simulation of brain perfusion images 10. Differences in the attenuation and scatter properties of such a material as compared with human tissue could be accounted for by appropriate image correction. Thus, the financial costs of implementing the technology can be minimal. SSP technology offers advantages of multiple different uptake patterns to be created for a single overall head size (i.e. for a single stack of attenuating slabs), as compared with conventional phantom designs that have fixed dimension cavities and can only reproduce a single uptake pattern.

Although a simplified patient template was used to generate phantom images in this study, it is straightforward to simulate more complex, subtle image features that are sometimes seen in clinical data. For example, ^123^I-FP-CIT patients often display increased tracer uptake in areas rich in serotonin transporters, such as the pons and thalamus 18. Reduced uptake is often also seen in the ventricles. These appearances may impact on semi-quantification results directly, particularly if a wide striatal region of interest is used (such as that described for the ‘Southampton’ method 19). They may also change the performance of the registration algorithm, which is a key part of many semi-quantification packages. Through its inherent flexibility, the SSP design enables investigation of such variability. For example, the ventricles defined in the anatomical labelling atlas could readily be incorporated into the current anatomical template.

In addition, SSP technology offers an ideal solution for validation of digital in-silico phantoms. The same anatomical template and assumed radionuclide concentration could be used for both the virtual and physical phantoms, offering benefits from direct comparison. In particular, recent work on simulated ^123^I-FP-CIT images 7 suggests that differences in striatal volume can give rise to differences in measured striatal binding ratios. The SSP could be used to confirm such results. Thus, the new SSP presented in this study has the potential to provide a meaningful contribution to the field of ^123^I-FP-CIT research.

## Conclusion

This paper has reported a simple ^123^I-FP-CIT phantom design based on subresolution sandwich phantom technology. The phantom uptake pattern was created from adaptation of the MNI template, registered to a 3D printed head structure based on CT. Each slice from this anatomical template was printed with a standard inkjet printer containing radioactive ink solution. The printed paper sheets were then interleaved between thin attenuating slabs, to create a full head phantom.

Reconstructed SPECT images of an assembled phantom (based on a striatum to reference brain count density ratio of 8 : 1) were assessed in terms of the striatal binding ratios and striatal linear dimensions. All results were found to be within the range of measured values for 22 clinical non-Parkinsonian patients. Phantom assembly time and detected count rate were acceptable. This work provides the foundation for the generation of a range of more clinically realistic physical phantoms.

## Supplementary Material

Supplemental Digital Content is available for this article. Direct URL citations appear in the printed text and are provided in the HTML and PDF versions of this article on the journal’s website, *www.nuclearmedicinecomm.com*.
